# Effect of Leucovorin (Folinic Acid) on the Developmental Quotient of Children with Down's Syndrome (Trisomy 21) and Influence of Thyroid Status

**DOI:** 10.1371/journal.pone.0008394

**Published:** 2010-01-11

**Authors:** Henri Blehaut, Clotilde Mircher, Aimé Ravel, Martine Conte, Veronique de Portzamparc, Gwendael Poret, Françoise Huon de Kermadec, Marie-Odile Rethore, Franck G. Sturtz

**Affiliations:** 1 Institut Jerome Lejeune, Paris, France; 2 Laboratory of Applied Mathematics, Agrocampus, Rennes, France; 3 National Academy of Medicine, Paris, France; 4 Department of Biochemistry and Molecular Genetics, University of Limoges, Limoges, France; University of Toronto, Canada

## Abstract

**Background:**

Seven genes involved in folate metabolism are located on chromosome 21. Previous studies have shown that folate deficiency may contribute to mental retardation in Down's syndrome (DS).

**Methodology:**

We investigated the effect of oral folate supplementation (daily dose of 1.0±0.3 mg/kg) on cognitive functions in DS children, aged from 3 to 30 months. They received 1 mg/kg leucovorin or placebo daily, for 12 months, in a single-centre, randomised, double-blind study. Folinic acid (leucovorin, LV) was preferred to folic acid as its bioavailability is higher. The developmental age (DA) of the patients was assessed on the Brunet-Lezine scale, from baseline to the end of treatment.

**Results:**

The intent-to-treat analysis (113 patients) did not show a positive effect of leucovorin treatment. However, it identified important factors influencing treatment effect, such as age, sex, and concomitant treatments, including thyroid treatment in particular. A per protocol analysis was carried out on patients evaluated by the same examiner at the beginning and end of the treatment period. This analysis of 87 patients (43 LV-treated vs. 44 patients on placebo) revealed a positive effect of leucovorin on developmental age (DA). DA was 53.1% the normal value with leucovorin and only 44.1% with placebo (p<0.05). This positive effect of leucovorin was particularly strong in patients receiving concomitant thyroxin treatment (59.5% vs. 41.8%, p<0.05). No adverse event related to leucovorin was observed.

**Conclusion:**

These results suggest that leucovorin improves the psychomotor development of children with Down's syndrome, at least in some subgroups of the DS population, particularly those on thyroxin treatment.

**Trial Registration:**

ClinicalTrials.gov, NCT00294593

## Introduction

The symptoms of Down's syndrome (DS) result from a trisomy of chromosome 21 [1], [2]. A gene dosage effect [3], [4], resulting from the presence of three chromosomes 21, rather than the usual two, is responsible for symptoms and mild to severe mental retardation [5], [6]. There is currently no effective way to reduce this gene dosage effect. However, some of the metabolic disturbances have been studied and could be targeted pharmacologically [2], [7]. The correction of these dysfunctions by treating DS patients with active molecules may improve the mental condition and quality of life of these patients.

**Figure 1:**
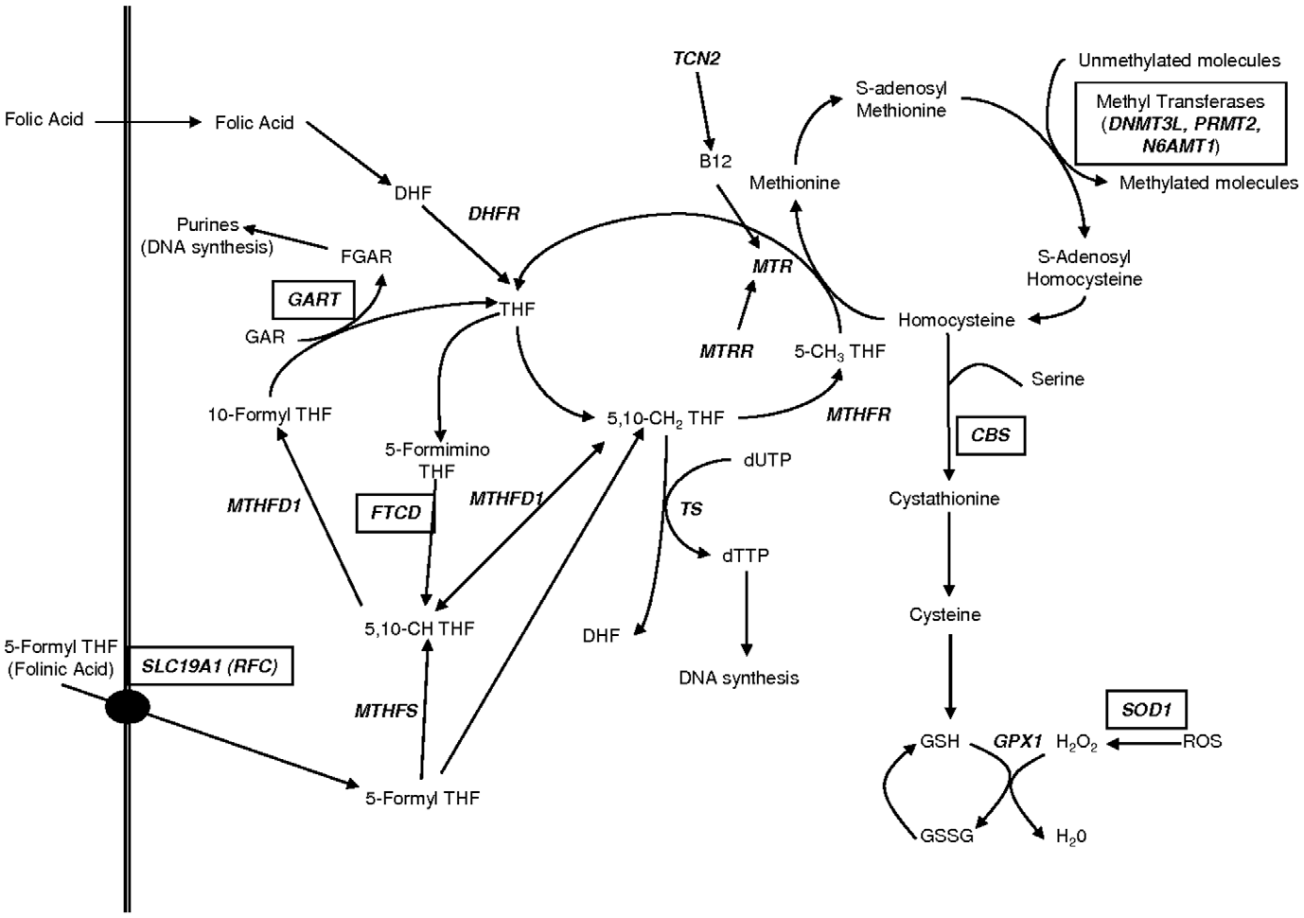
Folate metabolic pathways and methylation cycle. Gene names are shown in italics and according to the HGNC approved names. Genes in boxes are located on chromosome 21. Abbreviations used are: DHF: dihrofolate; THF: tetrahydrofolate; 5,10-CH2 THF: N5,N10-methylene THF; 5,10-CH THF: N5,N10-methenyl THF; 5-CH3 THF: N5-methyl THF; GAR: glycinamide ribonucleotide; FGAR: formyl-GAR; GSH: reduced form of glutathione; GSSG: oxidised form of glutathione; ROS: reactive oxygen species.

Folate deficiency is known to cause neurological, psychiatric and cognitive disorders [8], [9], [10], [11], [12], [13], [14], [15], [16], that can be reversed by folic acid administration [16], [17], [18]. DS probably involves either folate deficiency or defective folate use. Indeed, at least seven genes (SCL19A1, FTCD, GART, CBS, PRMT2, N6AMT1, DNMT3L with HGCN ID numbers: 10937, 3974, 4163, 1550, 5186, 16021, 2980, respectively) related to folate metabolism are located on chromosome 21 [19], [20]. The overexpression of these genes has been observed and causes a cascade of metabolic disturbances: low concentrations of folates, methionine, homocysteine, S-adenosyl methionine and serine and high levels of cysteine, cystathionine and methylated DNA (Figure 1) [7], [14], [21], [22], [23]. These disturbances partly account for the macrocytosis [24], glossitis and methotrexate hypersensitivity usually observed in these patients [25], [26], [27], [28], which can be partially corrected by folic acid treatment [27]. One previous study reported that folate treatment might improve the psychomotor development of DS patients [29], [30]. However, this study did not meet current standards for clinical trials and other studies did not confirm this finding [31], [32], [33]. Many DS children have since received empirical folate treatment.

We carried out a one-year, double-blind, placebo-controlled trial to assess the effect of folate supplementation on psychomotor development in young DS patients (3 months to 30 months of age). The registration number of this clinical trial was NCT00294593 (http://clinicaltrials.gov). Folinic acid/leucovorin (LV), also known as N5-formyl tetrahydrofolate (THF), was preferred to folic acid as it is already reduced. It can therefore enter the cytoplasm via the reduced folate carrier system (RCF), encoded by a gene (SLC19A1) located on chromosome 21. Leucovorin also bypasses the enzymatic steps of folic acid activation by DHFR, and is transformed by MTHFS and MTHFD1. Furthermore, leucovorin crosses the blood brain barrier more efficiently than folic acid resulting in greater bioavailability to neurons [34]. The clinical protocol took several factors potentially affecting developmental quotient into account, including age, sex and concomitant treatments. Hypothyroidism, which is frequent in DS patients, was carefully assessed as it may strongly affect mental development [35], [36].

## Materials and Methods

### Study Design

In this randomised, double-blind, placebo-controlled, single-centre clinical trial of leucovorin (LV), patients were randomised to two groups of equal size, one receiving placebo and the other leucovorin (LV 1 mg/kg daily per os). Treatment lasted 12 months, during which patients attended three scheduled visits: at inclusion (V_1_), after 6 months (V_2_) and after 12 months (V_3_) of treatment. All three visits included clinical examination, developmental evaluation and blood sampling (complete blood count, ferritin determination, thyroid check). Patients were assessed by a team of health professionals, including four physicians and six psychologists. The developmental test (main criterion) had to be carried out by the same psychometric examiner in each case. The protocol for this trial and the CONSORT checklist are available as supporting information (see Protocol S1 and Checklist S1). The study was conducted in accordance with the Helsinki Declaration and its amendments. It was approved by an institutional review board and by the French health authorities (AFSSaPS, CCPPRB Saint-Germain-en Laye). All parents gave written informed consent for the participation of their child. The informed consent statement and the informed consent form are provided as supporting information (see Informed Consent S1).

### Target Population and Inclusion Criteria

The inclusion criteria were: children aged 3 to 30 months, with complete, homogeneous trisomy 21. Patients had to weigh more than 4 kg, and be able to undergo assessment with the revised Brunet-Lezine psychometric scale (BLS). Patients with a history of leukaemia or any other condition that might interfere with evaluations of treatment efficacy, such as epilepsy or unstable hypothyroidism, were excluded.

### Sample Size

The sample size required was determined with nQuery Advisor® software (Statistical Solutions, Cork, Ireland). Based on internal unpublished data and a two-sided test with 80 percent power to detect a difference significant at the 5% level, with a standard deviation of 0.4, each group had to be composed of 47 patients for the detection of an expected 33% difference. Taking into account the probable frequency of non-evaluable patients, we planned to include 60 patients in each group.

### Outcome Measures

The primary endpoint (efficacy variable) was increase in global developmental age (DA), calculated as the following ratio: months acquired/months of follow-up. The revised Brunet-Lezine scale was used to assess developmental age [37], [38]. This scale of psychomotor development in early childhood investigates four fields of childhood development: (i) ‘posture’, (ii) ‘co-ordination’, (iii) ‘language’ and (iv) ‘sociability’, each with a corresponding subscore. The global DA score is derived from these four subscores. This scale has been validated for children of the general population with a developmental age of 2 to 30 months [38], [39]. It has been adapted for French populations and resembles the Bayley or Griffiths scales used in other countries.

### Safety

The safety of the treatment studied was assessed by recording adverse clinical events and carrying out biological assays at each visit. Any side effect arising during the trial, whether drug-related or not, was regarded as an adverse event and recorded. Sealed emergency decoding envelopes were available. Biological assays were analysed using paediatric reference ranges [40].

### Treatment and Randomisation

The drug was produced as 5 mg capsules, which had to be opened for administration—in a single intake—at breakfast. The active drug was administered as the pentahydrated calcium salt of leucovorin, to obtain a daily dose of 1.0±0.3 mg/kg. Drug compliance was assessed by counting the pills. The placebo and active capsules were obtained from the manufacturer of the drug (Folinoral®, Laboratoires Therabel Lucien Pharma, Levallois-Perret, France). Parents were asked not to administer any other folate-containing drugs to their children. Concomitant treatment was also minimised and noted in the case report form. Patients were assigned to a treatment by the random permuted block method (1∶1 ratio). Eligible patients were recruited as and when they were referred by participating physicians over a two-year period. An independent contract research organisation (LC2, Lyon, France) performed this operation and the randomisation list was not made available until the data were frozen. Thus, all investigators were blind to treatment assignment.

### Plan for Statistical Analysis

Data were encoded and input twice for quality assurance purposes. No interim analysis was planned. Data were frozen after double checking, recorded on a CD-Rom, and deposited at a lawyer's office before the code was broken. Data for patients who withdrew from the study early were analyzed for tolerability and, if possible efficacy. Demographic characteristics, clinical and biological tolerability and efficacy were analyzed by classical methods (Student's t test), using SPSS^©^ software (SSPS Inc. Chicago, IL, USA). A test of non linearity was planned for the Brunet-Lezine scale, using the data of children examined by the same person at all three evaluation points, V1, V2 and V3. We tested the nullity of the coefficient γ in the following equation: BL Global score = constant+α_i_+β×date+γ×date^2^, with α_i_, the effect on patient #i, β the date effect and γ the non linear date effect. The 3 evaluation points, V1, V2 and V3, were coded −1, 0 and 1, respectively. Once rejected the hypothesis of non-linearity of Brunet-Lezine scale over time, the Developmental Age (DA) was used to perform further statistical analysis.

In an exploratory way, a principal component analysis was carried out with SPAD^©^ software (Coheris, Courbevoie, France) [41]. This interesting method gives an axis that better summarizes the four DA variables (Global DA, Postural DA, Language DA, Coordination DA and Sociability DA). By projecting barycentres of the patients with same characteristics on the first axis, we identified influential cofactors. The factors included in the study were: leucovorin treatment, age at start of treatment, sex, body weight at start of treatment, developmental age at start of treatment (assessed by the Brunet-Lezine scale), treatment duration, thyroid treatment, psychometric examiner. Once the influential factors had been identified, several models of analysis of variance (ANOVA) were tested for treatment efficacy. We kept the ANOVA model that had good quality criteria (high variability of the Global DA explained by the model and low probability of simultaneous nullity of coefficients).

## Results

### Patient Population

**Figure 2:**
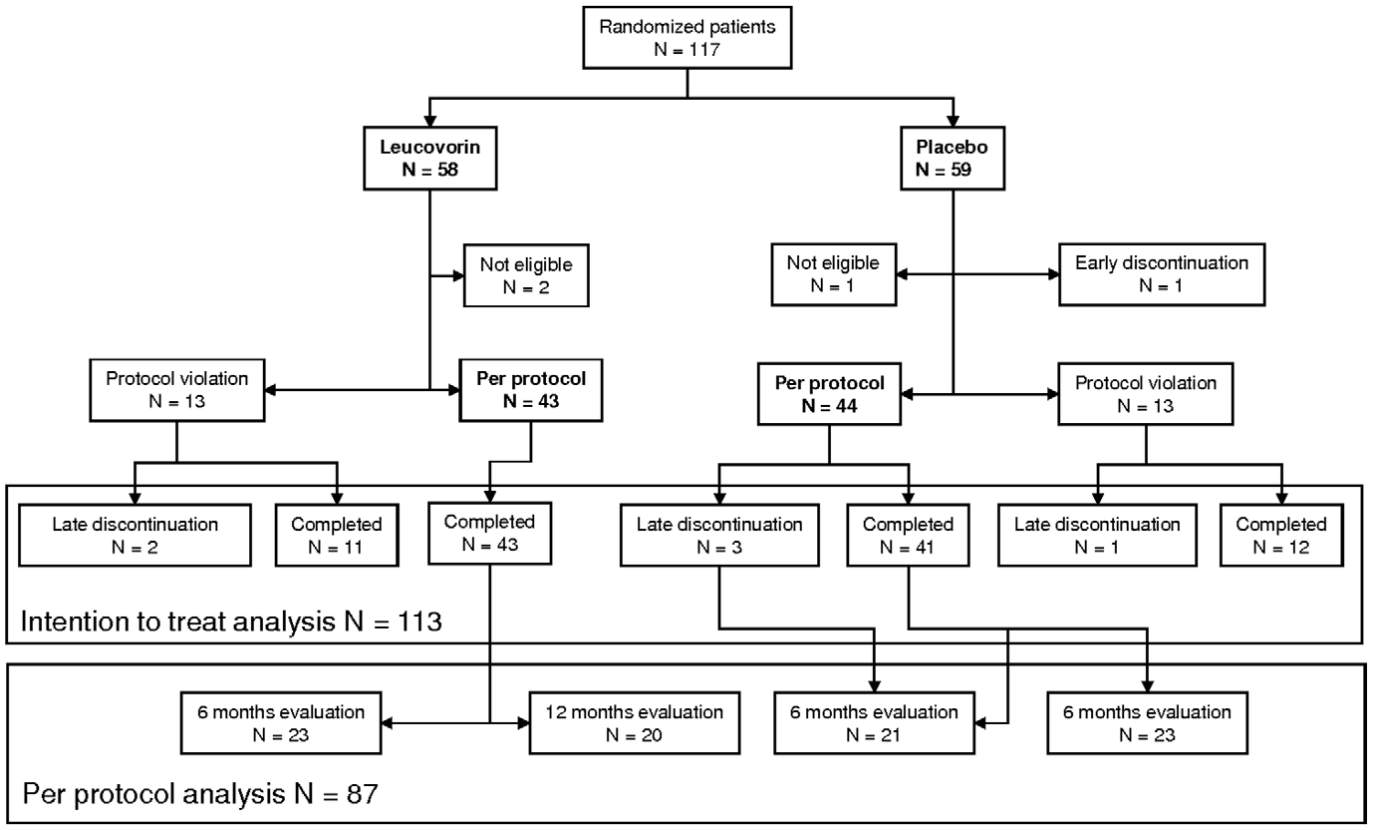
Flow chart of participants in the clinical trial. N = number of patients. Not eligible: patients with no efficacy and safety data after V_1_. Early premature V_1_ discontinuation: patients with no efficacy and safety data after V_1_. Late premature V_2_ discontinuation: patients not evaluated after V_2_ (6 months). Per protocol analysis: patients assessed by the same psychometric examiner during at least 2 visits. Major protocol violation: patients not assessed by the same psychometric examiner at 2 consecutive visits. Completed: patients without protocol violation and with 12 months of efficacy and safety data.

**Figure 3:**
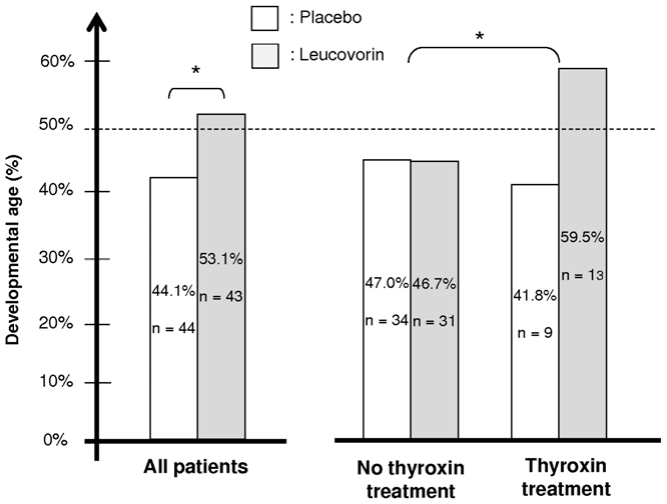
Effect of leucovorin treatment on change in global developmental age (DA). DA was measured for the patients receiving placebo or LV, in the per protocol population (87 patients) and as a function of a thyroxin treatment (N = number of patients, % change in developmental age with respect to a non-DS age-matched population).

All DS patients were recruited and included at the Jerome Lejeune Institute (Paris, France). Follow-up of the last participant ended 12 months later. In total, 117 patients were included in the trial (Figure 2). No patient withdrew due to treatment effects. Four of the 117 patients included withdrew early without an assessment of treatment efficacy. Thus 113 patients were included in the demographic and baseline description, and the safety and intention-to-treat analysis of efficacy. Evaluation by the same psychometric examiner before and after treatment, as required by the protocol, was not possible for 26 of the 113 patients (23.0%). The other 87 patients were included in an additional per protocol analysis of efficacy (Figure 3). This analysis took into account, for each child, the first and last evaluations by the same psychometric examiner (V_1_−V_3_ if possible, otherwise V_1_−V_2_ or V_2_−V_3_), after the linearity of the judgment criteria between V_1_−V_2_ and V_2_−V_3_ had been checked (data not shown).

### Demographic and Baseline Description

The baseline description recorded the medical history of patients, including Down's syndrome-related diseases in particular: cardiac abnormalities (persistent common atrioventricular canal, etc), digestive malformations (atresia), hypothyroidism requiring thyroxin treatment, ophthalmologic diseases (nystagmus, strabismus, cataracts). No history of haematological conditions was reported. The main demographic characteristics of the enrolled patients did not differ between the groups (Table 1). These demographic and baseline characteristics were similar in the per protocol population (Table 2).

**Table 1:** Demographic and baseline characteristics in the intention-to-treat group (113 patients).

Characteristics		n	LV group	Placebo group
Sex male/Female, n [%]		113	31 [55.4]/25 [44.6]	29 [50.9]/28 [49.1]
Birth characteristics, mean±SD^1^				
	Weight (g)	112	2.95±0.54	3.07±0.57
	Length (cm)	107	47.6±2.5	47.8±2.3
	OFP^2^ (cm)	103	32.9±1.7	33.2±1.4
Previous medical history, n [%]				
	Neonatal	108	6 [11.1]	8 [14.8]
	Cardiovascular	112	25 [45.5]	24 [42.1]
	Digestive	112	5 [9.1]	8 [14.0]
	Thyroid	112	14 [25.5]	10 [17.5]
	Ophtalmologic	112	5 [9.1]	8 [14.0]
General characteristics V_1_ 3, mean±SD [range]				
	Age (months)	113	14.4±8.1 [2.9–30.7]	13.7±8.9 [2.7–30.9]
	Weight (kg)	113	8.6±2.1 [4.5–14.0]	8.1±1.9 [4.81–12.00]
	Height/length (cm)	113	72.4±8.0 [56.0–85.5]	71.4±8.6 [56.0–89.0]
	OFP (cm)	113	43.4±2.5 [38.5–47.5]	43.2±2.3 [37.0–47.3]
Medical problems V_1_, n [%]				
	General	113	36 [64.3]	33 [57.9]
	Neurological	113	34 [60.7]	31 [54.4]
	Behavioural	113	7 [12.5]	7 [12.3]
BL^4^ age V_1_, *mean±SD [range] (months)*				
	Postural	113	8.6±4.6 [1.0–20.0]	8.4±5.0 [2.0–20.0]
	Co-ordination	113	8.8±4.7 [2.0–20.0]	8.0±4.7 [1.3–18.0]
	Language	113	8.1±4.1 [1.5–20.0]	7.7±4.2 [1.5–18.0]
	Sociability	113	8.5±5.0 [1.5–18.5]	8.2±5.0 [2.0–18.5]
	Global	113	8.6±4.5 [1.7–18.5]	8.1±4.6 [1.8–18.2]
DQ^5^ V1, *mean±SD [range] (%)*		113	62.6±13.4 [29.1–96.9]	63.4±13.5 [29.5–97.8]
Biology V_1_, *mean±SD*				
	Red cells (*10* 6 */mm* 3)	101	4.43±0.46	4.36±0.33
	Haemoglobin (*g/100 ml*)	100	12.75±1.31	12.62±0.86
	Haematocrit (*%*)	101	38.29±3.96	37.57±2.69
	MCV^6^ (*µ* 3)	101	86.54±4.59	86.19±4.26
	Platelets (*10* 3 */mm* 3)	100	378±116	402±114
	White cells (*mm* 3)	101	7437±2369	7157±2149
	Neutrophils (*mm* 3)	101	3135±1720	2953±1395
	Lymphocyte*s (mm* 3 *)*	101	3501±1288	3406±1298
	Monocytes (*mm* 3)	101	575±231	605±369
	Ferritin (*µg/l*)	90	50.61±66.55	47.63±56.54
	TSH^7^ (*mU/l*)	102	3.96±1.99	3.36±1.96
	Free T_4_ 8 (*pmol/l*)	94	15.44±3.14	15.28±2.80

1 Standard deviation.

2 Occipito-frontal perimeter.

3 inclusion visit.

4 Brunet-Lezine.

5 Developmental Quotient.

6 Mean corpuscular volume.

7 Thyroid-stimulating hormone.

8 Thyroxin.

**Table 2:** Demographic and baseline characteristics in the per protocol group (87 patients).

Characteristics		n	LV group	Placebo group
Sex male/Female, n [%]		87	23 [53.5]/20 [46.5]	25 [56.8]/19 [43.2]
Birth characteristics, mean±SD^1^				
	Weight (g)	86	2.96±0.58	3.18±0.56
	Length (cm)	83	47.6±2.8	48.1±2.3
	OFP^2^ (cm)	80	32.9±1.9	33.3±1.5
Previous medical history, n [%]				
	Neonatal	83	5 [11.9]	7 [17.1]
	Cardiovascular	87	22 [51.2]	17 [38.6]
	Digestive	87	3 [7.0]	8 [18.2]
	Thyroid	87	13 [30.2]	10 [22.7]
	Ophtalmologic	87	4 [9.3]	7 [15.9]
General characteristics V_1_ 3, mean±SD [range]				
	Age (months)	87	14.7±8.1 [2.9–28.4]	13.9±9.0 [2.7–30.9]
	Weight (kg)	87	8.76±2.10 [4.77–14.00]	8.22±1.92 [4.81–12.00]
	Height/length (cm)	87	72.7±7.9 [56.0–84.5]	71.7±8.4 [57.5–89.0]
	OFP (cm)	87	43.4±2.5 [38.5–47.5]	43.5±2.3 [37.0–47.3]
Medical problems V_1_, n [%]				
	General	87	25 [58.1]	26 [59.1]
	Neurological	87	24 [55.8]	25 [56.8]
	Behavioural	87	5 [11.6]	7 [15.9]
BL^4^ age V_1_, *mean±SD [range] (months)*				
	Postural	87	8.8±4.5 [1.0–20.0]	8.5±5.0 [2.0–20.0]
	Co-ordination	87	9.0±4.7 [2.0–19.0]	8.1±4.7 [1.3–18.0]
	Language	87	8.5±4.2 [1.5–20.0]	7.8±4.2 [2.0–18.0]
	Sociability	87	9.0±5.1 [2.0–18.5]	8.1±4.8 [2.0–18.5]
	Global	87	8.9±4.5 [1.7–17.6]	8.2±4.5 [1.8–18.2]
DQ^5^ V1, *mean±SD [range] (%)*			63.4±13.0 [29.1–91.3]	63.7±13.8 [29.5–97.8]
Biology V_1_, *mean±SD*				
	Red cells *(10* 6 */mm* 3 *)*	76	4.44±0.45	4.33±0.32
	Haemoglobin *(g/100 ml)*	76	12.82±1.21	12.50±0.76
	Haematocrit *(%)*	76	38.40±3.53	37.32±2.52
	MCV^6^ *(µ* 3 *)*	76	86.61±3.91	86.29±4.35
	Platelets *(10* 3 */mm* 3 *)*	76	371±106	401±123
	White cells *(mm* 3 *)*	76	7186±2172	7221±2237
	Neutrophils *(mm* 3 *)*	76	2908±1436	2968±1516
	Lymphocyte*s (mm* 3 *)*	76	3486±1388	3483±1333
	Monocytes *(mm* 3 *)*	76	582±217	580±270
	Ferritin *(µg/l)*	67	46.61±57.91	49.35±61.36
	TSH^7^ *(mU/l)*	78	4.06±2.05	3.42±2.11
	Free T_4_ 8 *(pmol/l)*	73	15.74±3.10	15.32±2.67

1 Standard deviation.

2 Occipito-frontal perimeter.

3 inclusion visit.

4 Brunet-Lezine.

5 Developmental Quotient.

6 Mean corpuscular volume.

7 Thyroid-stimulating hormone.

8 Thyroxin.

### Brunet-Lezine Scale Linearity

The psychometric scale used has been validated in a population of normal French children; the score obtained should increase linearly from 3 months to 30 months of age [38]. We checked the linearity of the scale in our DS population, in which children were evaluated three times (V1, V2 and V3) by the same psychologist. We obtained the following probabilities of nullity for each coefficient: probability (constant = 0) = 0, probability (β = 0) = 0, probability (γ = 0) = 0.698. We therefore rejected the hypothesis of non-linearity for BL global score.

### Efficacy Analysis

The intention-to-treat analysis (113 patients) revealed no greater positive effect of LV than of placebo on change in global DA (52.1% of normal DA vs. 51.5%, respectively). However, the per protocol multivariate analysis carried out on the 87 patients examined by the same person at the start and end of the trial showed that leucovorin probably enhanced DA. Children who received LV displayed a greater increase in DA (52.6%) than those treated with a placebo (42.8%, p = 0.046) (Figure 3). Cofactors were identified by principal component analysis on patients, for global DA score and the four subscores (posture, co-ordination, sociability, language). As we observed that the first axis was strongly correlated with Global DA (0.99), we concluded that it separated well children with high Global DA values from those with low Global DA values. The cofactors most strongly linked to the first axis (and thus global DA) were leucovorin treatment, age at start of treatment, sex and concomitant thyroxin treatment. These factors and their interactions with leucovorin treatment were thereafter assessed by analysis of variance (Table 3). The “age*thyroxin treatment” interaction cannot be studied because young children did not receive thyroxin treatment and the “sex*age” and “sex*thyroxin” interactions were not significant when added in the model.

**Table 3:** Factors influencing global developmental age in patients of the per protocol population (N = 87).

Factor	p	Significance	Degrees of freedom
Treatment with LV	0.0305	*	1
Sex	0.0064	*	1
Age (5 classes)	0.008	*	4
Thyroid treatment	0.3756	NS	1
LV x sex	0.4039	NS	1
LV x age	0.3016	NS	4
LV x thyroid treatment#	0.0348	*	1

The p values for each factor or interaction obtained in analysis of variance are shown. There were n−1 degrees of freedom, with n the number of possible levels of the factor.

# Patients that received both LV and Thyroxin treatment had a DA significantly higher than the other patients.

Patients who received LV displayed a greater increase in DA evolution (53.1%) than those on placebo (44.4%, p = 0.031) (Figure 3). Means were adjusted, to equilibrate the effect of the model. Two quality indicators showed that the selected statistical model for change in global DA was acceptable. The probability of simultaneous nullity of factors and interactions was 0.0007 and the model explained 36.4% of the variability of the main criterion. There was a significant interaction between LV treatment and concomitant thyroxin treatment (p = 0.035), with a DA of 59.5% recorded for patients receiving both leucovorin and thyroid treatment, versus only 41.8% for those receiving thyroid treatment only. The same model was computed for the four psychometric test subscales. Leucovorin also improved ‘co-ordination’ (56.5% vs. 42.18%, p = 0.019). It had a more pronounced effect on ‘co-ordination’ DA when the child was treated concomitantly with thyroxin (66.0% vs. 38.02%, p = 0.035). Similar trends were observed, but were not statistically significant, for ‘language’, ‘posture’ and ‘sociability’. These results were obtained when the population was split into five age groups. However, similar levels of significance were observed with two, three or four classes of age. Finally, mean change in global DA was greater for girls than for boys (53.6% versus 43.9%, p = 0.006) and girls showed larger improvements in ‘language’ and ‘sociability’ than boys (50.6% vs. 37.8%, p = 0.008 and 71.1% vs. 49.9%, p = 0.002, respectively).

### Compliance and Safety

Compliance was good, with patients taking 91% of their prescribed treatment. The mean duration of treatment was 358±44 days. DS patients present many common early childhood diseases, all of which were considered adverse events. We therefore recorded numerous concomitant treatments and adverse events. However no serious adverse event or drug-related adverse event was reported. For the 113 patients with safety data, a mean of 3.34 adverse events per patient occurred in the treated group, versus 3.84 in the placebo group (p>0.05) (Table 4). For the concomitant treatments, including drugs and rehabilitation, a mean of 7.8 treatments per patient were administered in the LV group, vs. 8.6 in the placebo group (p>0.05). No significant difference between therapeutic classes (acute toxicity class; ATC) was found (Table 5). No LV-related biological adverse events occurred during treatment and mean changes in biological data were similar between groups.

**Table 4:** Adverse events occurring in patients during the study period (113 patients).

Disease category	LV (56)	Placebo (57)
Infectious diseases	26	41
Hypothyroidism	1	1
Excitement, agitation	5	2
Eye and ear disease	23	31
Respiratory system diseases	116	122
Other diseases	16	22
**Total**	**187**	**219**

All events were regarded as adverse events and were assigned to a disease category.

**Table 5:** Number of patients who received concomitant treatments.

Treatments	LV (56)	Placebo (57)
Digestive tract and metabolism	32	36
Iron	17	11
Cardiovascular system	1	0
Dermatological	2	2
Corticosteroid	20	23
Systemic anti-infection agents	49	51
Non-steroid anti-inflammatory drugs	14	17
Analgesic drugs	22	27
Antiparasitic products	1	1
Respiratory system	33	40
Sensory organs	11	13
Rehabilitation	53	51
**Total**	**255**	**272**

## Discussion

Folates, including folic acid, in particular have been administered to DS patients based on a preliminary study conducted some time ago and not meeting current protocol standards [29], [30]. Assessments of the efficacy and safety of this treatment were required. Our intention-to-treat analysis showed no positive effect of leucovorin. However, the per-protocol analysis of a restricted group examined by the same psychologist at the beginning and end of trial revealed a positive effect of leucovorin. In the per-protocol analysis, the DA of DS children was significantly higher on leucovorin treatment than on placebo (53.1% vs. 44.4%, p = 0.031) (Figure 3). The per-protocol analysis also identified several cofactors, including thyroid treatment. DS patients receiving leucovorin and thyroxin treatment had a DA of 59.7%, whereas those receiving thyroxin only had a DA of 40.3% (p = 0.041).

The choice to use folinic acid/leucovorin rather than folic acid is debatable. Leucovorin may be a good alternative to folic acid because it is transported by the RFC (SLC19A1 gene) and efficiently transformed into 5,10-CH_2_ THF and 5-CH_3_ THF, which may be reduced by three methyl transferases—DNMT3L, PRMT2, N6AMT1—and by CBS activity (Fig. 1). Several studies have reported this molecule to have a higher bioavailability than folic acid [17], [34], [42]. No side effect of this drug was observed, despite its use at more than 10 times the RDA. Studies on folic acid have also reported an absence of adverse events [16]. As some positive effects were detected in our patients, we believe this dose of LV to be appropriate and safe for folate supplementation.

We also hypothesized that age at the start of folate supplementation might be important, as young neurons may be more sensitive to treatment, resulting in more efficient rescue from folate deficiency. We therefore enrolled very young patients in this study (3 months to 30 months). Our study population was representative of the general DS population in terms of birth weight, length, occipito-frontal circumference and malformation frequency [2]. However, the age range studied may have been too wide as major neurological acquisitions occur early in life and are highly variable. This may have resulted in heterogeneity of the population, potentially accounting, at least in part, for the negative result of the intention-to-treat analysis.

Several clinical trials had reported negative results regarding the effect of vitamin supplementation including folates [31], [32], [33], [43]. The results of these studies, although carefully monitored, may be challenged. Indeed, clinical trials on large DS populations are not easy to perform, as centres specialising in DS treatment do not have large patient cohorts. This is a major drawback of all studies. By contrast, the most recent study [43] included 156 patients with Down's syndrome and a mix of 5 antioxidants or folinic acid was given in a blinded manner. Folinic acid was not found to be active on cognitive functions. However, we believe the daily dose of folinic acid (0.1 mg vs. 1.0±0.3 mg/kg in our study) was too low to be truly active. The negative results of our intent-to-treat analysis may be due to our lack of expertise in managing large trials. Psychometric analysis is a critical factor in such trials. When we first wrote the protocol, we thought it would be possible for the same psychometric examiner to carry out all three evaluations (V1, V2 and V3). However, as some of our patients came from far away and had limited flexibility in terms of appointments, it was sometimes difficult for the same psychologist to carry out assessments at the beginning and end of the study (12 months later). As even well trained professionals may vary in terms of the running of tests, we believe that analyses in which the same psychologist carried out at least two of the tests (per protocol analysis) are likely to be more accurate, although this violates the intention-to-treat analysis protocol. These pitfalls should be carefully avoided in subsequent studies, and the use of internationally validated scales would be required for international trials. However such scales are not always available, may be complicated to use, may result in young DS patients systematically obtaining scores towards the bottom of the scale or may not be linear over long periods. The Brunet-Lezine scale has several advantages: it is well established, easy to use and reproducible. However, we will probably use international scales, such as the Griffith's scale in future studies. Nevertheless, the development of reliable scales remains a key issue.

The relationship between leucovorin and thyroxin treatment was not expected, although thyroid status is known to be important for the mental development of patients with trisomy 21. Several hypotheses may be proposed to explain the possible synergistic effect of LV and thyroid treatment. The first hypothesis is that the antioxidant effect of folinic acid may have been amplified by the oxidative stress induced by thyroxin treatment. Indeed, thyroid hormone has been shown to increase mitochondria metabolism and thus production of reactive oxygen species (particularly in patients with hypothyroidism) and to induce an antioxidant imbalance [44], [45], [46]. Furthermore, thyroid hormone has been shown to down regulate the expression of a gene involved in the catabolism of free oxygen radicals, superoxide dismutase-1 [47]. All together, these facts may explain why thyroxin may have stimulated the cognitive development of the children and how folinic acid may have limited the thyroxin drawbacks linked to oxidative stress.

The second hypothesis is that homocysteine, which is involved in both the folate pathway and thyroid status, may play a key role in the relationship between LV and thyroid treatment. Homocysteine is remethylated or leaves the cycle by being further transformed by CBS (cataplerosis) (Figure 1). Thyroid hormones probably induce the expression of certain genes, but these results must be interpreted with caution, given the complexity of the different metabolic pathways. Plasma total homocysteine (tHcy) concentration is inversely correlated with thyroid hormone levels [48], [49] and returns to normal levels when euthyroidism is restored [50], [51]. Hypothyroid patients also have low plasma folate concentrations, and folate supplementation in addition to thyroid treatment is recommended [48]. A relationship between thyroid status and folates has also been shown for methyl tetrahydrofolate reductase (MTHFR) activation in humans and rats [52], [53], [54]. Some studies have reported that IQ is related to tHcy concentrations in DS patients, and to polymorphisms of two enzymes of the remethylation pathway—MTHFR (C677T) and a transcobalamin (TCN C776G) [22]. Unfortunately, tHcy levels and MTHFR, TCN2 and MTHFD1 polymorphisms were not investigated in our patients. Future studies should consider these factors, to improve our understanding of therapeutic mechanisms. However, these findings suggest that homocysteine may play a negative role in the neurons of DS patients alone or via its metabolites [12], [55].

The improvement of cognitive functions associated with LV treatment may increase the autonomy of the DS population. If maintained over time, it could increase quality of life and may have economic implications. A follow-up study is planned for these patients at the age of seven years, to check whether the LV-induced enhancement of development is maintained over time. The interaction between folates and thyroxin also requires investigation. Further studies should also focus on the cofactors identified in this trial. Despite technical difficulties, this trial strongly suggests that the psychomotor development of DS patients may be improved by treatments acting on metabolic pathways. These encouraging results should lead to a search for specific drugs targeting the metabolic disturbances induced by the overexpression of chromosome 21 genes.

## Supporting Information

Protocol S1 Trial Protocol.
(0.39 MB DOC)

Informed Consent S1 (0.03 MB DOC)

Checklist S1 CONSORT Checklist.
(0.19 MB DOC)
