# Supplementary material for: Effect of Leucovorin (Folinic Acid) on the Developmental Quotient of Children with Down's Syndrome (Trisomy 21) and Influence of Thyroid Status
Source: PLoS One. 2010 Jan 11;5(1):e8394. doi: 10.1371/journal.pone.0008394 (PMC2799517; doi:10.1371/journal.pone.0008394)
Supplement: Protocol S1 — Trial Protocol. (0.39 MB DOC) [file pone.0008394.s001.doc]

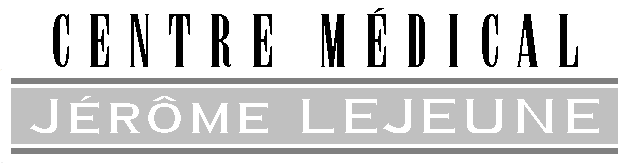


CLINICAL RESEARCH PROTOCOL
CONFIDENTIAL

Title **PHASE II-III CLINICAL TRIAL OF ONE YEAR'S DURATION, CARRIED OUT AS A DOUBLE-BLIND PLACEBO-CONTROLLED STUDY IN TWO PARALLEL GROUPS, WITH EVALUATION OF THE EFFECTS OF FOLINIC ACID (1 mg/kg/day) ON COGNITIVE FUNCTION IN YOUNG CHILDREN WITH DOWN'S SYNDROME.
ENTRAIN – A PHASE II-III TRIAL, WITH DIRECT INDIVIDUAL BENEFIT.**

Stage Protocol completed 15th January 2000 and submitted to AFSSaPS

Sponsor Centre Médical Jérôme Lejeune (CMJL)
 Hôpital Notre Dame de Bon Secours
 68, rue des Plantes Tel. + 33 1 45 45 55 56

75014 PARIS Fax + 33 1 45 45 13 43

Principal investigator, co-ordinator
 Professor Marie-Odile RETHORÉ
 Centre Médical Jérôme Lejeune
 Hôpital Notre Dame de Bon Secours
 68, rue des Plantes Tel. + 33 1 45 45 55 56

75014 PARIS Fax + 33 1 45 45 13 43

# PROTOCOL SUMMARY

***Sponsor*** Centre Médical Jérôme Lejeune

***Active agent***  Calcium salt of folinic acid, pentahydrated

***Title*** Phase II-III clinical trial of one year's duration, carried out as a double-blind placebo-controlled study in two parallel groups, with evaluation of the effects of folinic acid (1 mg/kg/day) on cognitive function in young children with Down's syndrome. ENTRAIN — a phase II-III trial with direct individual benefit.

***Co-ordinator*** Professor Marie-Odile RETHORé

***Study centre*** Centre Médical Jérôme Lejeune
68, rue des Plantes Tel. +33 1 45 45 55 56
75014 PARIS Fax +33 1 45 45 13 43

***Start date of the trial*** January 2000

***Phase and duration of the trial*** Phase II-III study to confirm efficacy. Inclusion period of 1.5 years.

***Aims*** The principal aim of the study is to evaluate, in a double-blind, placebo-controlled study, the effects of folinic acid on cognitive function in young children with Down's syndrome. The secondary aims of this trial are to evaluate the tolerance of this product in this population and to assess its possible effects on behavioural problems.

***Methodology*** Double-blind, placebo-controlled trial with two parallel groups.

***Number of subjects*** 100 (50 on folinic acid, 50 on placebo).

***Diagnosis, inclusion criteria*** We will include children with Down's syndrome in a stable state and old enough for assessment with the Brunet-Lezine test (revised version; 6 to 24 months), after obtaining written informed consent from the parents or guardians of these children, who must be resident in France and contactable by telephone. We will not include patients with a history of myeloproliferative syndrome, infantile seizures (current), or patients who have started thyroid treatment in the last three months or who are taking antifolic acid treatments (methotrexate, phenytoin). All patients presenting myeloproliferative syndrome or requiring thyroid treatment will be excluded from the trial.

***Treatment studied*** Folinic acid per os (Folinoral®) 1 mg/kg/d, or placebo, formulated as capsules.

***Evaluation criteria*** Principal criterion: change in developmental age (DA).
Secondary criteria: clinical and biological tolerance, opinion of the parents (concerning the continuation of treatment), opinion of the doctor and psychologist.

***Statistical methods*** Student's t tests (or Mann-Whitney U tests) for quantitative variables and χ^2^ tests for qualitative variables. PCSM® (Deltasoft) and SPSS® software will be used, together with SAS if required.
Stratification will be carried out retrospectively, based on initial development quotient.

# ABBREVIATIONS AND DEFINITIONS

## AbBreviations

0 concerns the initial examination

DA developmental age

GCP good clinical practice

C consultation

CBS cystathionine β-synthase

IRB institutional review board

CGI clinical global impression

CMJL Centre Médical Jérôme Lejeune

VAS visual analogue scale

f concerns the final examination

i  concerns an intermediate examination

I item

d day

kg kilogramme

mg milligramme

NA not applicable

ND not determined

CC cranial circumference

PO, po per os

SOP standardised operating procedure

DQ developmental quotient

IQ intellectual quotient

W week

SAM S-adenosyl methionine

SOD superoxide dismutase

t concerns intermediate telephone contacts

T21 trisomy 21 (Down's syndrome)

THF tetrahydrofolate

## DEfinitions

Folinic acid C_20_H_23_N_7_O_7_              Molecular mass: 491.5.


folinate, 5‑formyl tetrahydrofolic acid, 5‑formyl THF
N‑[[[(amino‑2 formyl‑5 hydroxy‑4 tetrahydro‑5,6,7,8 pteridinyl‑6)               methyl] amino]‑4 benzyl] L‑(+) glutamic acid.

Calcium folinate: pentahydrated calcium salt of folinic acid
C_20_H_21_CaN_7_O_7_.5H_2_O
Molecular mass: 601.6.

Folinoral® Pharmaceutical formulation used in this study. Its active ingredient is the pentahydrated calcium salt of folinic acid. The section from the Vidal guide concerning this product from Laboratoires Thérabel-Lucien Pharma is provided in the appendix.

Brunet-Lezine In this study, developmental age, the principal judgement criterion, is determined by means of the revised Brunet-Lezine test. This very standard test is distributed by EAP (Editions et Applications Psychologiques, 95 boulevard de Sébastopol, 75002 PARIS, France). It is described in the section dealing with judgement criteria and a sheet corresponding to this test is supplied in the appendix.

# BACKGROUND

Down's syndrome is a dysmorphic syndrome associated with mental retardation and is caused by trisomy of chromosome 21, detectable by karyotyping.

## GENERAL INFORMATION

Diseases caused by chromosomal abnormalities are unusual in that the genetic abnormality is purely quantitative — repetition of part of the information present on the chromosome in the case of trisomies. Several observations confirm the integrity and quality of the information contained in aneuploid genomes. In particular, half the children of mothers with Down's syndrome have the same trisomy as their mother, whereas the other half have a normal karyotype and 46 chromosomes. The normal phenotype of this second group of children demonstrates that the maternal chromosomes 21 are qualitatively normal and that the presence of two copies of this chromosome is sufficient to cause the disease in the other children.

Thus, trisomy leads to excessive protein synthesis rather than to the synthesis of abnormal proteins. Its effect is therefore one of gene dose. These metabolic abnormalities being quantitative rather than structural, treatment may be possible, as most drugs function precisely by modifying the rates of enzymatic reactions.

In addition, a foetus presenting several serious metabolic abnormalities is highly unlikely to survive, as in many cases a single serious abnormality is lethal in itself. For this reason, only a small number of the metabolic abnormalities associated with the 700 genes present on chromosome 21 need to be taken into account in Down's syndrome. Changes in the rate of only a handful of chemical reactions in the brain may make it possible to improve considerably the cognitive functions of patients with Down's syndrome.

Treatment is thus potentially possible.

## Down's syndrome

Down's syndrome is the most frequent cause of mental retardation, accounting for one third of all cases. The incidence of Down's syndrome is fairly stable and is of the order of one case in every 700 children. In Alsace, the incidence of Down's syndrome is one case per 1050 live births [[[1]](#endnote-2)]. Paradoxically, prenatal diagnosis has not led to a decrease in the number of births of children with Down's syndrome, which has indeed remained stable or even increased [[[2]](#endnote-3)]. Over the last 30 years, maternal age has increased consistently, and maternal age has clearly been identified as the most important risk factor [[[3]](#endnote-4),[[4]](#endnote-5),[[5]](#endnote-6)]. In 1930, children with Down's syndrome had a life expectancy of nine years; their life expectancy had increased to 55 years in 1986, and is undoubtedly now greater than 60 years. There are currently about 40,000 to 50,000 patients with this condition in France, and several million such patients worldwide. The socio-economic burden represented by these patients is considerable, because a non-negligible proportion of these patients are institutionalised (costing several hundreds of thousands of francs per year). An improvement, even if only slight, in the IQ of patients with Down's syndrome, might allow a larger proportion of these patients to find work and would increase their autonomy, allowing them to live independently, thereby greatly reducing health expenditure.

## Folates and down's syndrome

The principal role of folates in the body is to supply single-carbon radicals during transmethylation reactions. Many lines of evidence suggest that folates play a key role in the pathogenesis of mental retardation in patients with Down's syndrome:

- Macrocytosis in these patients
- The location of the reduced folate transporter gene (SLC19A1-hFOLT1) on chromosome 21
- The role of folates in two of the reactions of *de novo* purine synthesis, one of which depends directly on the product of the *GART* gene, which is also located on chromosome 21
- The role of the serine residue of folates in the remethylation of homocysteine to produce methionine. By contrast, the reaction to generate cystathionine involves the production of S-adenosyl methionine, which is catalysed by cystathionine β‑synthase, encoded by a gene on chromosome 21.
- The existence of a functional deficiency of acetylcholine, a neuromediator synthesised through folate-dependent methylation reactions.

All genetic diseases affecting folate metabolism are associated with low intelligence. In addition, many diseases associated with low intelligence are accompanied by genuine problems with monocarbon metabolism: sarcosinaemia, glycinaemia, homocysteinuria, hisidinaemia, carnosinuria, hyperprolinaemia, hyperhydroxyprolinaemia, iminodipeptiduria, Hartnup disease and Lesch-Nyhan syndrome.

In adults, folate deficiency causes recurrent haematological problems and infections, but may also cause neurological problems (polyneuropathies, restless leg syndrome), together with cognitive and psychiatric problems (depression, dementia); these disorders may be readily reversed by treatment [[[6]](#endnote-7)], confirming the important role of folates in the functioning of the central nervous system. However, adult patients may not display intra-erythrocyte folate deficiency or macrocytosis [[[7]](#endnote-8)]. Folate administration significantly improves the results obtained in Wechler's psychometric assessments of memory and IQ in patients with mental problems [[[8]](#endnote-9)].

The importance of folates in the development of the central nervous system is also clearly demonstrated by the key role of folate deficiency in neural tube malformation, with even minor deficiencies increasing the risk of such malformations [[[9]](#endnote-10)].

Conversely, the importance of folates in Down's syndrome is clearly demonstrated by the deleterious effects of drugs with antifolate activity in these patients: sulfamides (e.g. trimethoprime-sulfamethoxazole), antiepileptic drugs (phenytoin) and antimitotic agents.

An open initial clinical trial of folinic acid has been carried out using a cohort of 69 patients with Down's syndrome between the ages of 1 and 44 years. The inclusion and judgement criteria were psychotic disorders and Alzheimer-type dementia. This study demonstrated a beneficial effect of treatment in more than 50% of patients. No adverse effect was recorded, with the exception of one case of excitation, which regressed when the dose was decreased. The dose administered was between 0.15 mg/kg/d and 1.92 mg/kg/d. The dose regularly having a therapeutic effect was about 1.00 mg/kg/d. Confirmation is required, as there was no control group in this study, but the existence of a dose-dependence effect strongly suggests that this effect is real [[[10]](#endnote-11)].

All these elements are consistent with Down's syndrome patients having cerebral folate deficiency or displaying a defect in folate use in certain cells of the central nervous system. These problems may be closely related to the low intelligence of these patients, and their correction may improve cognitive function.

## folic and folinic acid

For higher function problems, such as psychoses, folinic acid has been shown to be more effective than folic acid for the administration of folates. There are several reasons for this: it is more stable, its digestive absorption is greater, it has a longer half-life and it crosses the blood-brain barrier more readily. Indeed, folic acid cannot pass from the blood into the central nervous system without prior transformation and is eliminated by an active transport system [[[11]](#endnote-12),[[12]](#endnote-13)].

## Conclusion

It would therefore seem necessary to test the hypothesis that folates may affect the cognitive functions of patients with Down's syndrome, under certain conditions:

- The product used should be folinic acid, which is the most likely to be effective. It should be used at a sufficiently high dose to ensure that any effect is detectable;
- The study should be rigorous in terms of its methodology (control group, double-blind, placebo-controlled etc.) and its quality (quality criteria recommended by GCP, use of standardised operating procedures, auditing etc.);
- The judgement criteria must be validated;
- Folinic acid is extremely well tolerated in general, but tolerance monitoring is required in this particular population;
- Finally, the detection of an effect on cognitive function will require the treatment of young children whose brains do not yet have developmental abnormalities too fixed and too irreversible for treatment and whose brains are still capable of growth and development.

# practical information

We provide here details of the people involved in this trial and the site at which the research will take place. The *curriculum vitae* of the participants are provided in the appendix.

## Sponsor

Centre Médical Jérôme Lejeune
68, rue des Plantes Tel. +33 1 45 45 55 56
75014 PARIS Fax +33 1 45 45 13 43

## site of the research

Centre Médical Jérôme Lejeune (CMJL)
Hôpital Notre Dame de Bon Secours
68, rue des Plantes Tel. +33 1 45 45 55 56
75014 PARIS Fax +33 1 45 45 13 43

A full description of the Centre Médical Jérôme Lejeune is provided in the appendix.

## trial participants

### Principal investigator-Co-ordinator

Professor Marie-Odile RETHORÉ Tel. +33 1 45 45 55 56
Centre Médical Jérôme Lejeune Fax +33 1 45 45 13 43
68, rue des Plantes Home tel. +33 1 43 26 10 68
75014 PARIS Mobile: +33 6 11 07 01 13

### Co-investigators

Doctor Clotilde MIRCHER
Doctor Martine CONTÉ
Centre Médical Jérôme Lejeune
68, rue des Plantes Tel. +33 1 45 45 55 56
75014 PARIS Fax +33 1 45 45 13 43

Anne-Marie BAUD, psychologist
Emmanuelle BELTRAM, psychologist
Babak SHAFEE, psychologist
Centre Médical Jérôme Lejeune
68, rue des Plantes Tel. +33 1 45 45 55 56
75014 PARIS Fax +33 1 45 45 13 43

### Nurse

Tatiana LAPENNE
Centre Médical Jérôme Lejeune
68, rue des Plantes Tel. +33 1 45 45 55 56
75014 PARIS Fax +33 1 45 45 13 43

### Monitor, methodology, biometrics

Doctor Henri BLEHAUT Tel. +33 1 45 45 55 56
Centre Médical Jérôme Lejeune Fax +33 1 45 45 13 43
68, rue des Plantes Home tel: +33 1 47 22 68 41
75014 PARIS Mobile: +33 6 14 12 51 17
  E-mail blehauth@aol.com

### Industrial logistics partner

Laboratoires Thérabel-Lucien Pharma
represented by Doctor Frédéric MAS
Directeur Médical
15, rue de l’Hôtel de Ville Tel. +33 1 46 43 90 90
92200 NEUILLY-SUR-SEINE Fax +33 1 46 43 90 99

# aims

The principal aim of this phase II-III trial with direct individual benefit, ENTRAIN, is to carry out a double-blind, placebo-controlled evaluation of the effects of folinic acid on cognitive function in young children with Down's syndrome.

The secondary aims of this trial are to evaluate the tolerance of this product in this defined population and to assess its possible effects on behavioural problems.

# design of the trial

## summary

The chronology of the trial is summarised below in table form.

|  | Initial consult | Bimonthly contacts | Intermediate consult | Final consult |
| --- | --- | --- | --- | --- |
|  | C0 | Ct | C1 | C2 |
| **Date** | 0 months |  | 6 months | 12 months |
| **General** |  |  |  |  |
| Information of the parents | **+** | - | - | - |
| Consent | **+** | - | - | - |
| Inclusion criteria | **+** | - | - | - |
| Non-inclusion criteria | **+** | - | - | - |
| History | **+** | - | - | - |
| **Clinical examination** |  |  |  |  |
| Medical examination | **+** | * | **+** | **+** |
| Height-weight-CC | **+** | - | **+** | **+** |
| Doctor's opinion (CGI) | - | - | **+** | **+** |
| Psychologist's opinion (VAS) | - | - | **+** | **+** |
| Brunet-Lézine Test | **+** | - | **+** | **+** |
| Parents' opinion of treatment | - | - | **+** | **+** |
| **Biological tests** |  |  |  |  |
| Blood formula | **+** | * | **+** | **+** |
| Thyroid assessement | **+** | * | **+** | **+** |
| Iron, Ferritin | **+** | * | + | + |
| Adverse effects | **+** | + | + | + |
| Date of the next visit | **+** | + | + | - |

* only if required

## trial description and chronology

The total duration of follow-up for any given patient in the trial will be 1 year ± 30 days.

The first medical consultation will include:

- Information about the trial and the signing of the consent form;
- Confirmation of the diagnosis, checking for the presence of karyotyping data in the patient's medical records;
- Checking the inclusion and non-inclusion criteria, including, in particular, ability to undergo the revised Brunet-Lezine test for determining developmental age (DA);
- A general clinical and neurological examination, with measurement of height, weight and CC;
- Prescription of routine biological tests (blood formula, iron, ferritin, thyroid assessment) and other specific examinations, if necessary.

The first consultation with the psychologist will include a psychological examination and the revised Brunet-Lezine test.

Intermediate contact will be made with the parents at intervals of no more than two months. During these intermediate contacts by telephone, the interview by a doctor from the CMJL will focus particularly on the tolerance of folinic acid treatment.

If necessary, the final consultation may take place early, in cases of withdrawal from the study.

The intermediate consultation (6 months ± 15 days) and the final visit (1 year ± 30 days) will include:

- A general clinical and neurological examination, with measurement of height, weight and CC;

- The recording of any adverse events;

- The recording of the doctor's opinion concerning the child (CGI);

- The recording of the overall opinion of the psychologist concerning the development and behaviour of the child on a visual analogue scale (VAS);

- The revised Brunet-Lezine test (determination of DA);

- The recording of the parents' opinion on the treatment of the child;

- The prescription of routine biological tests (blood formula, iron, ferritin, thyroid assessment) and other specific examinations if required.

The entire study will therefore include only standard examinations: three medical examinations, three psychological examinations and three sets of standard biological tests. Between consultations, three to five telephone contacts are planned to help the family and for the monitoring of tolerance and compliance with treatment. If possible, all the examinations will take place on the same day, with the same doctor and the same psychologist.

The total duration of the inclusion period will be 1.5 years.

# subject selection

Patients will be selected on the following criteria:

## inclusion criteria

Patients with the following characteristics will be included:

- Down's syndrome (free and homogeneous trisomy 21),

- Suitability for assessment with the revised Brunet-Lezine test at the time of the first examination and (predictively) the second examination. Children should thus be aged between about six and 24 months,

- Weight between 6 and 26 kg,

- Planned stable maintenance treatment for the next year,

- Good compliance likely and possibility of carrying out subsequent examinations within the required time frame,

- Patient resident in France

- Parent or guardian of the child contactable by telephone,

- Parent or guardian having received and understood the information document and signed the informed consent form.

## non-inclusion criteria

Patients presenting the following characteristics will not be included:

- History of myeloproliferative syndrome, transient myeloproliferative disorder, leukaemoid reaction or neonatal thrombocytopenia,

- Current infantile seizures, unbalanced epilepsy,

- The initiation of thyroid hormone treatment in the three months preceding the trial (patients on stable treatment with thyroid extracts[[13]](#footnote-2)* for more than three months may be included),

- Patients presenting folinic acid intolerance.

## exclusion criteria

Patients will be removed from the study if one of the following criteria is met:

- Occurrence of myeloproliferative syndrome;
- Initiation of thyroid hormone treatment during the study (patients already on stable thyroid extract treatment for more than 3 months may be included);
- Folic or folinic acid treatment (other than that studied) for more than two months during the trial: Alvityl®, Azedavit®, Azinc®, Carencyl®, Cernevit®, Elevit®, Elvorine®, Folinate de calcium, Folinoral®, Forvital®, Léderfoline®, Lofenalac®, Osfolate®, Perfolate®, Plenyl®, Quotivit®, Soluvit®, Spéciafoldine®, Synergyl®, Tardyféron®, Vivamyne®, Zédène®,
- Treatment with a drug with antifolate activity for more than two months of the year (methotrexate, phenytoin).

For patients withdrawing early from the trial, as much information as possible will be obtained by telephone and the final examination will be carried out as soon as possible (within a month of withdrawal).

# treatments

## product studied: folinic acid

Folinic acid will be administered in the form of Folinoral® (calcium folinate), formulated appropriately for this trial. The characteristics of Folinoral® can be found in the monograph from the Vidal guide provided in the appendix.

### Presentation, formulation and labelling

The labelling and presentation of the product will conform to the legislation in force (Article R5123 of the French Public Health Code).

The product will be presented in the form of capsules to be taken *per os*. These capsules will in no case be administered in this form. They will always be opened and their content mixed with some cold food (yoghurt, compote, jam) to prevent any possible degradation, although the product is stable. Folinic acid is almost tasteless.

The capsules of the active product will contain 5 mg of folinic acid in the form of the pentahydrated carbon salt of N‑[[[(amino‑2 formyl‑5 hydroxy‑4 tetrahydro‑5,6,7,8 pteridinyl‑6) methyl] amino]‑4 benzyl] glutamic acid and excipients.

The placebo will be presented in a strictly identical manner and will be composed of inactive excipients.

The parents will be provided with the treatment on the day of the first consultation and on the day of the intermediate consultation. On both occasions, they will be provided with enough capsules for 6.5 months (196 days) of treatment (about 50% of the patients are from outside the Parisian region). All of the blister packs of treatment, whether empty or full, must be brought to each consultation.

### Dose and duration of treatment

The dose used will be 1.0±0.3 mg/kg/d at the start of treatment. This dose is relatively high, to ensure that no effect is missed. Folinic acid is non-toxic. No overdose has been observed, even during the administration of very high doses. Indeed, once the amount required has been used, the excess is eliminated (see the section from the Vidal provided in the appendix).

The table below shows the dose to be administered as a function of weight.

WEIGHT (kg) DOSE (mg) No. CAPSULE

These doses are high because the dose in mg/kg is likely to decrease as the child grows during the observation period. At the ages studied, the children are unlikely to put on more than 3 kg per year, so the dose should never fall below 0.7 mg/kg. The dose could be increased as a function of weight, without exceeding 20 mg/d, at the time of the intermediate consultation or telephone contact. Any such change in dose will be recorded in the "treatment and rehabilitation" section of the observation notebook.

In cases of functional problems, such as irritability or hyperactivity, the dose may be progressively decreased, by one capsule per day, by the investigator. If the symptoms persist or are too severe, the investigator could stop the treatment early. If possible, the patient should be re-evaluated at a consultation before the treatment is stopped.

The total duration of treatment will be 1 year ± 1 month. The product will be administered as a single intake at breakfast.

### Compliance

If an intake of folinic acid is forgotten, it should not be administered at the time of the next intake.

Compliance will be assessed retrospectively by counting the number of capsules remaining at the end of treatment. The parents will bring all the blister packs of capsules, whether full or empty, to each consultation for this purpose. If the parents forget to bring the treatment to the consultation, they will be asked to send the blister packs by post.

### Storage and distribution

The treatment will be stored in a safe place, at room temperature or under refrigeration.

A stock-taking form will be completed by the CMJL, in the form of a table. This form will facilitate distribution of the correct treatment to the correct patient and the return of unused medication. At the end of each course of treatment, unused products will be counted for the determination of compliance by the CMJL, before being destroyed.

### Randomisation

Prospective randomisation, based on the use of random number tables, will be applied. Randomisation will be carried out in blocks to prevent imbalance between the two groups. This randomisation will be carried out by an organisation independent of the Centre Médical Jérôme Lejeune.

### Emergency decoding

A set of envelopes making individual decoding possible will be stored at the Centre Médical Jérôme Lejeune. Treatment decoding will be allowed only if knowledge of the treatment administered is liable to modify subsequent treatment. In this trial, there should therefore be no need to open the emergency decoding envelopes.

However, in cases of emergency decoding, the name and signature of the person responsible for the decoding will be noted on the decoding envelope and in the observation notebook, together with the date, time and reason for decoding and action taken as a result. The opened decoding envelope will be placed in the pocket of the observation notebook. The time of decoding will mark the end of the observation period for the subject concerned.

In no case will these envelopes be used for decoding at the end of the trial.

## associated treatments

Other treatments will be avoided during the study. However, if necessary, they will be authorised and noted in the observation notebook, except for treatments with folic or folinic acid (other than the product studied) for more than two months during the trial, which will lead to exclusion from the trial.

# adverse effects

All adverse events occurring during the trial will be described by the investigator in the observation notebook, whether or not they are related to the treatment studied: nature, severity, duration, frequency, reversibility, cessation of treatment. An assessment of the extent to which the event can be attributed to the treatment will be recorded. If treatment is stopped for a minor adverse event or the implication of folinic acid is unclear, a rechallenge test will be carried out, if possible.

Intercurrent illnesses occurring during the trial will be treated as adverse events.

If an adverse event is present at the end of the study, the investigator will transmit reports relating to its progression to the study monitor until this event is cured or stabilised.

Adverse events will be considered serious if they place the subject's life in danger, lead to death, require or prolong hospitalisation or cause disability or significant temporary or permanent clinical incapacity. Serious adverse events will be reported immediately to the monitor (within 24 hours, by telephone) who will declare them to the AFSSaPs. Other adverse events must be notified to the monitor within eight days.

Written confirmation, on a form for recording adverse events, must be sent to the monitor within three days of the occurrence of a serious adverse event.

If a serious or highly unpleasant adverse event is attributed to the treatment studied, the treatment will be stopped and symptomatic treatment initiated.

All new information relating to this research and with implications for patient safety must be declared, in the form of a toxic or unexpected effect declaration, to AFSSaPS, and should be communicated to the investigators.

# data collected and evaluation criteria

## data collection

Information relating to each patient will be noted in the observation notebook, using a black ballpoint pen, during the course of the study. Each page of the observation notebook will be dated and initialled or signed by the investigator. The investigator may delegate completion of this notebook to another authorised person, provided that he or she checks and initials the entries. The people authorised to complete the observation notebook are those members of the CMJK listed above in the practical information section.

## functional and clinical data

The following will be carried out at each consultation (C_0_, C_i_ and C_f_):

- General physical examination, with measurement of height, weight and cranial circumference (CC);
- Neurological examination, including the systematic examination of abnormal movements, nystagmus, protrusion of the tongue, tremors, tonus, static position, walking, osteotendinous reflexes and sphincter control;
- The revised Brunet-Lezine test, assessing developmental age (DA) [[[14]](#endnote-14)]. Photocopies of the results will be rendered anonymous and included in the observation notebook.
- Collection of information about associated treatments.

The following items will be assessed at consultations C_1_ and C_f_:

- CGI in the middle and at the end of treatment [[[15]](#endnote-15)];
- Psychometric improvement, as assessed by psychologists using a VAS;
- Tolerance, with particular emphasis on neurological tolerance.

## paraclinical examinations

These examinations will be carried out at all consultations, at six-month intervals: determination of blood formula, iron and ferritin concentrations and thyroid assessment. Photocopies of the results will be rendered anonymous and included in the observation notebook.

## missing data

If a patient does not return for examination after a year of treatment, the data for the intermediate examination will also be treated as those of the final examination. If no data are collected after the initial examination, these data will be considered missing for the analysis. Reasons for leaving the trial early will systematically be sought, via telephone interviews at least, to check that there is no link between withdrawal from the trial and the treatment (particularly as concerns tolerance).

## evaluation criteria

### Tolerance of folinic acid

All adverse events will be recorded, whether or not they appear to be related to the treatment. Clinical and biological tolerance will be evaluated:

- Clinical tolerance will be assessed on a case-by-case basis, with dose adjustment if required.
- Biological tolerance will be assessed by monitoring changes in biological variables. Case-by-case follow-up will be carried out, using the results of the first laboratory tests after the initiation of folinic acid treatment as reference. These data will also be used to assess the significance of changes in biological test data for all the patients treated.

### Efficacy

Efficacy will be evaluated using several criteria.

The principal evaluation criterion will be the change in developmental age, the profile of which is known for patients with Down's syndrome [[[16]](#endnote-16)]:

Translation for the figure: Age mental = mental age; Age reel = real age.

Developmental age will be assessed by the revised Brunet-Lezine test administered before (DA_0_) and after (DA_f_) treatment: (DA_f_ - DA_0_). The revised Brunet-Lezine test is widely recognised as a test for the evaluation of psychomotor development in infants. It has been validated and its metrological qualities (sensitivity, fidelity-homogeneity, dispersion and validity, including for Down's syndrome children) are well known. For further information about this test, please consult reference 13.

The other criteria for efficacy evaluation will be:

- CGI, as recorded by the doctor;
- A score for psychologist satisfaction, estimated on a VAS, evaluating the opinion of the psychologist on the subjective improvement of the child since the start of treatment.
- The response of the parents to the question "If possible, would you like to continue this treatment ?" The response will be scored as follows: in favour of continuing treatment/against continuing treatment/does not know or no opinion expressed.

# losses to follow-up and premature withdrawal from the trial

The reasons for which a subject may be withdrawn from the trial are as follows:

- Demand from the patient, withdrawal of consent;
- Clear aggravation;
- Occurrence of a serious or unpleasant adverse effect associated with the product tested;
- Poor compliance
- Occurrence of myeloproliferative syndrome;
- Initiation of thyroid hormone treatment during the trial (patients already on thyroid extracts treatment for more than 3 months may be included);
- Treatment with folic or folinic acid (other than the product studied) for more than two months during the trial.

The reasons for the treatment being stopped early or for premature withdrawal from the trial should be documented and reported clearly in the observation notebook, on the appropriate page. All cases of premature cessation of treatment or withdrawal will be analysed in terms of tolerance and efficacy, provided that it is possible to obtain the appropriate data.

The number of subjects lost to follow-up should be minimised and as much information as possible should be collected for these subjects (by postal or telephone contact, for example).

# data processing and statistics

All the variables will be subject to statistical analysis, but any adverse effects will also be analysed on a case-by-case basis.

## number of cases

The number of subjects required has been determined with nQuery Advisor® software. Calculations have been carried out for developmental quotient, based on previous data and the following assumptions for calculation of the number of subjects per group:

Two-tailed Student's t tests with equal numbers in the two groups

α risk 0.05

Mean improvement on placebo over one year 0.666 years

Mean improvement on folinic acid over one year 0.900 years

Standard deviation 0.4 years

Power (1-β) 80%

Two-group t test (identical numbers of subjects each group); power (puissance); number of subjects per group

If we adopt a power of 80%, as is usual for phase II-III studies, we require 47 subjects per group (94 in total). We have therefore decided to recruit 50 subjects per group, giving 100 complete observations in total.

An intermediate statistical analysis will be carried out without decoding for the first 60 subjects, to check that the variances of the two groups are equal. The number of subjects required will be recalculated at this point. If the number of subjects must be changed, an amendment to the protocol will be required.

## data input, decoding and analysis

A double-input system will be used before decoding. Once the data have been cleaned in this way and the base has been corrected, the database will be frozen. Decoding will not take place until this has been achieved.

The analysis will concern the patients receiving the product and will include:

- An analysis of the study population for the principal descriptive variables: age, sex, selection criteria, maternal and paternal ages, term, birth weight, birth height, CC at birth, APGAR score and, at each consultation, height, weight, CC, examination, treatment, DA, DQ, CGI, parental opinion etc.,
- An analysis of clinical tolerance,
- An analysis of biological tolerance (blood formula, iron, ferritin, thyroid assessment) based on statistical methods (changes in biological features between C_0_ and C_f_), analysing all data outside the normal range,
- An analysis of efficacy based on the variables defined above, with retrospective stratification for initial DQ.

## Tests used

Quantitative data will be analysed by Student's t tests (or ANOVA). For statistical analyses of laboratory test results, data may be adjusted before the analysis, according to JAMA norms [[[17]](#endnote-17)]. At the end of treatment, for quantitative data also available at the start of treatment (height, weight, CC, DA, biological test results), tests will be carried out to assess the change in the item considered under treatment (I_f_-I_0_) rather than on the absolute value of the item after treatment (I_f_). If the data are non-normally distributed and it is impossible to normalise them by an appropriate transformation, they will be analysed with the non-parametric Mann and Whitney U test.

For qualitative variables, Pearson's χ^2^ test will be used for comparisons, with Fisher's exact test used if sample size is considered insufficient.

Statistical analysis will be carried out by the monitor from the CMJL, using PCSM® (Deltasoft) and SPSS® software. If necessary, assistance will be sought from a specialist organisation (SAS software).

# ethical issues

This trial will be carried out in accordance with:

- The recommendations of the Helsinki Declaration, provided in the appendix
- Good clinical practice, as defined by the International Conference on Harmonisation (CHPH/ICH/135/95, adopted on 17/07/1996)
- The Huriet Law (dating from 20/12/1988), as amended by the laws of 23/01/1990 and 25/07/1994 and their decrees of application
- The law of 01/07/1994 relating to the processing of nominative data collected for health research, amending the *Loi Informatique et Libertés* (Computing and Liberty Law) dating from 06/07/1978.

## Good clinical practice

This study will be carried out according to the standardised operating procedures (SOP) of the Centre Médical Jérôme Lejeune. These procedures are available as a manual.

## ethics committee, Irb

The Co-ordinator will submit this protocol to the IRB of Saint-Germain en Laye Hospital, by recorded post (with notification of delivery), providing the information required by article R.2029 of the French Public Health Code.

All modifications to the protocol must be submitted to this IRB for approval in cases of substantial modification or for notification in cases of minor modification, and will be communicated to the AFSSaPS if necessary.

## Qualifications

The medical director of the centre is Dr Marie-Odile Rethoré, Professor of Genetics and Member of the Academy of Medicine. She is clearly competent to co-ordinate this trial. The doctors of the centre have considerable experience in the field of mental disability and have attended additional, appropriate training. They have also already worked as investigators in clinical drug trials. The psychologists routinely carry out evaluations of the patients followed at the CMJL. The knowledge and experience of the personnel involved in this trial are therefore consistent with the research proposed.

The *curriculum vitae* of the participants in this trial are supplied in the appendix.

## adequacy of the research site

The Centre Médical Jérôme Lejeune has accreditation from the French Ministry of Health as a referral centre for the follow-up of diseases of chromosomal origin affecting intelligence. Down's syndrome patients account for 75% of the patients followed at the CMJL. This centre has accreditation for biomedical research without direct individual benefit, covered by authorisation number 10435S, issued on April 8^th^ 1999.

The facilities of the CMJL include an infirmary with an emergency care kit specially designed for clinical trials. This centre is located within Notre Dame de Bon Secours Hospital, which has an emergency department. If required, the nearest available intensive care unit is that run by Professor Carlet at Saint Joseph Hospital, 800 metres away. This site is therefore entirely appropriate for this clinical study, particularly given that tolerance to folinic acid is considered to be excellent: according to the Vidal guide (see appendix), folinic acid is non-toxic; no overdose has ever been observed, including during the administration of very high doses. Indeed, once the amount required by the body has been used, the excess is eliminated.

## Information of the patients and informed consent

The legal representative (parent or guardian) of the child will be informed of the aims of the research, its duration, the methods used, the advantages and possible constraints and risks. This information will be provided by the investigator, in the form of an information sheet given to the patient's legal representative. This information sheet is provided in the appendix.

Written informed consent will be obtained from the subject's legal representative. The text of the consent form is provided in the appendix. The presence of a signed consent form will be checked during monitoring of the trial, whilst respecting medical secrecy. Consent forms will be stored in sealed envelopes at the CMJL and will be archived at the end of the study.

## Anonymity

All individuals involved in the running of this trial will be required to respect professional secrecy. In addition, the anonymity of the patients will be maintained. In France, these requirements are laid down in article R.5120 of the Public Health Code.

Finally, data processing will be carried out in accordance with the laws in force, including the Law "*Informatique et Libertés*" dating from January 6^th^ 1978 and its decrees of application.

# practical considerations

## duration of the trial

The total duration of the inclusion period will be 1.5 years.

## monitoring

Regular telephone contact (at least every two months) will be established, in addition to consultations with investigators and/or the monitor and/or the nurse at the CMJL. For data checking, direct access to the source data for the patients (medical files) will be required and will be supplied by the investigator to allow the monitor to make comparisons with the data collected in the observation notebook.

## Audit

Audits of the trial and of files relating to the trial will be carried out before the trial, at the start of the trial and after the trial, before decoding, by PRA International-ARCAM (16 rue de Courcelles, 75017 Paris). Other audits may also be carried out.

## recording and archiving of data

Data will be recorded legibly, with a black ballpoint pen, in the observation notebook. The missing data will be clearly indicated as ND (not determined) or NA (not applicable).

Corrections made before the separation of duplicate entries will be made by crossing out the incorrect data such that they remain legible. The correction will then be written alongside the original data, signed and initialled. Errors detected after the separation of duplicate entries will be corrected using dedicated sheets in which the initial data and the corrected data will be noted, together with the date and the investigator's signature.

The investigator and the sponsor must keep the essential documents relating to this trial (source data, originals of the informed consent forms) for at least two years after the end of the last authorisation for market release obtained in an ICH region and until there are no more demands for market release pending or envisaged in an ICH region.

## final report, publication, confidentiality

This trial may not be the subject of any written or oral communication (publication, communication) without the agreement of the CMJL (article R.5120 of the French Public Health Code).

In case of publication, the order of the authors will be determined amicably, on principle as follows: the investigating doctors, the monitor, the psychologists and the co-ordinator. Laboratoires Thérabel-Lucien Pharma will be acknowledged at the end of the article.

# administrative procedures

These procedures will respect the legislation in force in France.

## insurance

The guarantees of the insurance contract covering civil responsibility taken out by the CMJL will apply, in accordance with the Law of December 20^th^ 1998, particularly as concerns article L.209-7 of the Public Health Code (civil responsibility contract for the sponsors of biomedical research taken out with an insurance company specialising in health risks: SM Biomédic, ZA de Luscanen Ploeren, BP220, 56006 Vannes cedex).

## DEclaration of intent to the AFSSaPS

A letter of intent will be sent to the AFSSaPS (*Agence Française de Sécurité Sanitaire pour les Produits de Santé*) by recorded post (with notification of delivery), by the sponsor, and will include the details required by article  R.2032 of the French Public Health Code.

## Information of the directors of health establishments

A letter of declaration will be sent by the monitor to the Director of the Centre Médical Jérôme Lejeune, including the details required by article R.5124 of the French Public Health Code.

## Information of pharmacists of health establishment

NA.

## financial conventions

No financial conventions have been signed concerning this trial.

# DOCUMENTS supplied in the appendix

-  VIDAL guide entry concerning Folinoral®

- Helsinki Declaration

- Information sheet (Art. L.209-9 of the French Public Health Code) and informed consent form (Art. L.209-9 of the Public Health Code)

- Example of completed revised Brunet-Lezine test

- *Curriculum vitae* of the study participants

- Presentation of the CMJL

# responsibilities of the sponsor and investigators

*The responsibilities of the Centre Médical Jérôme Lejeune (CMJL, sponsor) are:*

- Preparation of the material required for the trial and setting up the trial;
- Monitoring of the running of the trial, the end-of-trial consultation, checking the observations notebooks, checking that informed consent has been obtained for all subjects, data input and analysis.

*The responsibilities of Laboratoires Thérabel-Lucien Pharma are:*

- Communication to the CMJL of all new information concerning the tolerance of the product;
- Confidentiality and respect for the publication rules concerning this trial;
- Supplying the product.

*The responsibilities of the investigators are:*

- Storage in a safe place of all documents, products and materials associated with this trial;
- Informing the subjects and obtaining freely given, written, informed consent;
- Availability for the trial;
- Respect for the anonymity of the subjects;
- Completion of observation notebooks, carrying out the trial according to the protocol and notification of adverse effects within the specified delays;
- Provision of information to the families concerning the results of the work.

Date: Date:

Prof M.-O. RETHORÉ, sponsor (CMJL) Dr H. BLÉHAUT, monitor (CMJL)
investigator and co-ordinator

Date: Date:

Dr C. MIRCHER, investigator (CMJL) Dr F. MAS (Thérabel-Lucien-Pharma)

Date:

Dr M. CONTÉ, investigator (CMJL)

# references

1. Stoll C, Alembik Y, Dott B, Roth MP. Epidemiology of Down syndrome in 1188 265 consecutive births. Am J Med Genet 1990;suppl 7:79-83. [↑](#endnote-ref-2)
2. Hoshi N, Hattori R, Hanatani K, Okuyama K, Yamada H, Kishida T, Yamada T, Sagawa T, Sumiyoshi Y, Fujimoto S. Recent trends in the prevalence of down syndrome in Japan, 1980-1997. Am J Med Genet 1999;84:340-345. [↑](#endnote-ref-3)
3. Hook EB, Fabia JJ. Frequency of Down syndrome by single-year maternal age interval: Results of a Massachusetts study. Teratology 1978;17:223-228. [↑](#endnote-ref-4)
4. Hook EB, Chambers GM? Estimated rates of Down's syndrome in live-births by study - inplication of the "risk" figures for genetic counseling and cost-benefit analysis of prenatal diagnosis programs. In Bergsma D, Lowry RB, Trimble BK, Feingold M (eds): Numerical taxonomy on birth defects and polygenic disorders. Birth Defects Original Series 1977;13:124-141. [↑](#endnote-ref-5)
5. Hook EB, Linnsajo A. Down's syndrome in live births by single-year maternal age intervals in a swedish study: Comparison with results from New-York state. Am J Hum Genet 1978;30:19-27. [↑](#endnote-ref-6)
6. Enk C, Hougaard K, Hippe E. Reversible dementia and neuropathy associated with folate deficiency 16 years after partial gastrectomy. Scand J Haematol 1980;25:63‑66. [↑](#endnote-ref-7)
7. Botez MI, Cadotte M, Beaulieu R, Pichette LP, Pison C. Neurobiologic disorders responsive to folic acid therapy. Can Med Assoc 1976;115(3):217‑223. [↑](#endnote-ref-8)
8. Botez MI, Botez T, Léveillé J, Bielmann P, Cadotte M. Neurophychological correlates of folic acid deficiency: facts and hypotheses. In Folic acid in neurology, psychiatry and internal medecine. Botez MI, Reynolds EH eds. Raven Press NY 1979:435‑461. [↑](#endnote-ref-9)
9. Christensen B, Arbour L, Tran P, Leclerc D, Sabbaghian N, Platt R, Gilfix BM, Rosenblatt DS, Gravel RA, Forbes P, RozenR. Genetic polymorphisms in methylenetetrahydrofolate reductase and methionine synthase, folate levels in red blood cells, and risk of neural tube defects. Am J Med Genet 1999;84:151‑157. [↑](#endnote-ref-10)
10. Lejeune J, Rethoré MO, de Blois MC, Peeters M. Psychose infantile, syndrome pseudo-Alzheimer, et trisomie 21. Thérapie 1989;44:115‑121. [↑](#endnote-ref-11)
11. Brocker P, Bonhomme P, Lods JC. Folates : de la déficience à la carence. Rev Gériatr 1990;15(8):365-373. [↑](#endnote-ref-12)
12. Spector R. Cerebro fluid folate and the blood-brain barrier. In Botez MI, Reynold EH: Folic acid in neurology, psychiatry and internal medecine. Raven Press New-York 1979:187. [↑](#endnote-ref-13)
13. ^^ Levothyroxine : 25 µg ± 50% [↑](#footnote-ref-2)
14. Josse D. Brunet-Lézine révisé, échelle de développement psychomoteur de la première enfance. Editeur EAP (Établissements d’Applications Psychométriques) Paris 1997; 307 pages. [↑](#endnote-ref-14)
15. Von Frenckell R. Impressions cliniques globales. In : JD Guelfi, L’évaluation clinique standardisée en psychiatrie ; Pierre Fabre:93‑97. [↑](#endnote-ref-15)
16. Lejeune J. Pathogénie de la débilité de l’intelligence dans la trisomie 21. Ann Génét 1991;34(2):55-64. [↑](#endnote-ref-16)
17. Tables des valeurs en unités classiques et unités du système international des différents paramètres et composés physiologiques et pharmaceutiques. JAMA janvier 1991, hors série. [↑](#endnote-ref-17)
