# Supplementary material for: Effect of Leucovorin (Folinic Acid) on the Developmental Quotient of Children with Down's Syndrome (Trisomy 21) and Influence of Thyroid Status
Source: PLoS One. 2010 Jan 11;5(1):e8394. doi: 10.1371/journal.pone.0008394 (PMC2799517; doi:10.1371/journal.pone.0008394)
Supplement: Informed Consent S1 — (0.03 MB DOC) [file pone.0008394.s002.doc]

**ENTRAIN: a double-blind versus placebo clinical trial to evaluate the effect of folinic acid on young Down's syndrome patients**

**Informed consent statement**

The number of treatments available for your child is still very limited. For this reason, we are proposing that you participate in the evaluation of a medication, folinic acid. Indeed, we think that this medication might improve the development of your child. This medication is often prescribed to children of his (her) age or to adults with other diseases. It is well tolerated and numerous trisomic patients who have received it, tolerated it well.

In order to objectively judge the efficacy of this product, it will be necessary to randomize the treatment of your child. He (she) will receive either an inert product with the same appearance but without any pharmacological activity, or folinic acid. Neither your physician, nor you will know the nature of the treatment.

This study will last one year. During this year, your child will have a medical consultation once every 6 months (3 times: at the start, after 6 months, after 12 months). During this year, he will receive this treatment once daily. The product will not be administered as pills but will have to be opened with scissors and the content mixed with cold food (apple compote, jam, yogurt). The treatment will be given to you by the physician at the Medical Center Jerome Lejeune. For each medical visit, you will have to bring back the treatment packages (blisters), empty or full of pills.

During the consultations, your child will be examined as usually done by the physician, including for development and the biological examinations will be the same as usually performed on children of this age (blood tests, iron and ferritine, thyroid hormones). During the study, you will be regularly contacted by staff members of the Medical Center Jerome Lejeune in order to check the tolerance of the treatment. In case of need and after having talked to your physician, the treatment might be decreased or stopped. The physicians of the Medical Center Jerome Lejeune will be reachable by your current general practitioner to answer any question he may have regarding the trial.

Throughout the study, we ask you to inform us of any change you must make regarding other medications and we ask you to not give any medication that may interfere with the product tested during the trial: Azine, Calcium folinate, Folinoral, Lederfoline, Lofenalac, Osfolate, Perfolate, Scluvit.

This research has been properly authorized by the CCPPRB (Consulting Committee for the Protection of People in Biomedical Research) of Saint-Germain-en-Laye to which it has been submitted. A specific insurance has been contracted by the Medical Center Jerome Lejeune.

At the end of the trial, your physician will inform you regarding the results and the usefulness of the treatment. You may ask any questions you want. Of course, you are entirely free to decline to participate in the trial or to remove your child from the study any time. This would absolutely not interfere with the quality of care he (she) will receive. According to the law of December 20, 1988 related to people's protection in biomedical research, you are asked to sign the following consent form.

**Consent**

I, hereby (Name, First name) ….

Legal representative of (Name, First name) …

Living at: (address)…

Give my agreement to his (her) participation in the trial ENTRAIN organized by the Medical Center Jerome Lejeune "a double-blind versus placebo clinical trial to check the effect of folinic acid given to young patients affected by Down's syndrome".

I fully understood the information provided above. If I agree, I will retain all my rights warranted by law. My agreement does not undermine the responsibility of the organizers.

The computer program used for compiling the data of the study has received an authorization by the CNIL, in accordance with the Article 40-1 under the law "Computers and freedom". The medical data regarding my child, as well as his (her) living habits will be used only by staff members of the Medical Center Jerome Lejeune for the good of the study, or by the Authorities in case of requests and under conditions insuring medical confidentiality. At any time, I may exert my right to have access to the data or correct it by contacting Dr Bléhaut at the Medical Center Jerome Lejeune.

At any time, I may obtain information from the physician who will sign below. If I find it preferable, I may end the participation of my child in the study and this will not interfere with the quality of care my child will receive.

Date and signature of the legal representative Name and signature of the physician
